# Multicenter evaluation of preoperative and standalone embolization in meningiomas

**DOI:** 10.3389/fonc.2025.1626753

**Published:** 2025-11-26

**Authors:** Duan Yu, Guohui Huang, Jun Shen, Yanan Li, Jie Xu, Ziwei Xu, Jian Li, Dongwei Dai

**Affiliations:** 1 Huadong Hospital, Fudan University, Shanghai, China; 2 Changhai Hospital the Second Military Medical University, Shanghai, China

**Keywords:** intracranial meningioma, preoperative embolization, hybrid surgery, standalone embolization, angiographic devascularization, multicenter retrospective cohort

## Abstract

**Background:**

Preoperative embolization has been proposed to reduce intraoperative blood loss and facilitate meningioma resection, however its clinical utility remains debated. This multicenter study evaluated the safety, efficacy, and angiographic outcomes of preoperative embolization and standalone embolization in intracranial meningiomas.

**Methods:**

In this retrospective cohort from January 2017 to January 2022, patients were stratified into three groups: standalone embolization (SE), combined preoperative embolization and craniotomy (hybrid surgery, HS), and craniotomy alone (control). Tumor characteristics, procedural metrics, and clinical outcomes were compared.

**Results:**

Compared to control group, the HS group exhibited significantly larger tumors (68.8 ± 10.6 cm³ vs 35.7 ± 11.3 cm³, *P*<0.001) but achieved reduced intraoperative blood loss (9.8 ± 2.3 mL/cm³ vs 19.2 ± 6.5 mL/cm³, P<0.001) and higher gross total resection rates (70.1% vs 46.2%; P = 0.025). Compared to HS group, the SE cohort had smaller tumor volume (24.7 ± 5.2 cm³ vs 68.8 ± 10.6 cm³; *P*<0.001), better baseline neurological function score (median mRS 0 vs 1; *P*<0.001), and showed higher total devascularization rate (56.3% vs 25.4%; *P* = 0.008) after embolization procedure. Tumors in SE group were supplied exclusively by the external carotid artery (ECA). At a median 24-month follow-up, recurrence rates and neurological change were no different across groups.

**Conclusion:**

Hybrid surgery optimized surgical resection for large meningiomas by reducing blood loss and improving resection completeness, while standalone embolization demonstrated feasibility for select small tumors with ECA. Both two strategies showed acceptable safety and effectiveness.

## Introduction

The inconsistency in the role of preoperative embolization for meningioma treatment is evident, with no reports of embolization as a standalone treatment plan. The main advantages of primary embolization include reducing intraoperative blood loss and softening solid tumors through post-embolization necrosis (1–3). However, significant heterogeneity exists in clinical outcomes (4–6).

In clinical practice, technical variabilities, including selection of target feeding arteries, extent of embolic devascularization, and management of high-risk anastomoses, critically impact the efficacy and safety of embolization. Advances in neurointerventional technologies, such as innovative microcatheters and liquid embolic agents, now enable more precise and controlled tumor devascularization (7), raising the possibility of curative intent single-session embolization for select cases, particularly small, deep-seated meningiomas with favorable vascular anatomy. Notably, embolization as a standalone therapy has not been systematically evaluated.

This multicenter study aims to compare outcomes of three treatment strategies, including hybrid surgery (HS group, preoperative embolization followed by craniotomy), standalone embolization and control group, treated by craniotomy alone. We analyzed clinical outcomes, tumor characteristics and procedural metrics to evaluate the safety, efficacy, and appropriate indications for each approach.

## Materials and methods

This study was designed as a retrospective observational analysis. From January, 2017 to January, 2022, the patients with meningioma in two neurosurgery centers, were enrolled. All patients were informed of details of this study, including allowing anonymous disclosure of clinical data and images, and signed an informed consent.

Inclusion criteria: 1. Diagnosed by pathologically in HS group and control group; 2. Two senior radiologists independently and simultaneously determined that it was a meningioma based on the results of MRI scan in SE group; 3. Over 20 years old; Exclusion criteria: 1. Recurrence of meningioma; 2. Multiple meningiomas; 3. After radiotherapy; 4. Loss to follow-up.

### Study design

This multicenter retrospective cohort study from January 2017 to January 2022 enrolled patients with intracranial meningiomas from two neurosurgical centers. The study protocol received institutional review board approval (HD20170111), and all participants provided written informed consent for anonymous use of clinical data and imaging.

Inclusion Criteria: 1. HS and control groups: confirmed meningioma diagnosis histopathologically; 2. SE Group: MRI-based diagnosis confirmed independently by two board-certified neuroradiologists. 3. Age ≥20 years at intervention. Exclusion Criteria: 1: Recurrent or multiple meningiomas; 2. History of prior radiotherapy; 3. Incomplete follow-up data (<6 months).

### Preoperative DSA

After being diagnosed with meningioma, the angioarchitecture was defined by DSA in SE or HS group. This angiography looked for main tumor feeding arteries, extracranial–intracranial anastomosis, extent of tumor blush, and draining veins.

According to the dominant supplying arteries of patients in SE group and HS group, we divided them into three types 1: External carotid artery (ECA) system predominant, 2: Internal carotid artery (ICA) system predominant; 3. Vertebrobasilar artery (VA) system predominant. In the control group, due to the absence of routine cerebral angiography, the supplying arteries were not assessed.

### Embolization

Following administration of heparin, an 8 F femoral artery sheath was arranged. A 6 long sheath (80 or 90 cm) was advanced to the origin of the common carotid artery or VA, while a 5 guiding catheter (115cm or 125cm) was positioned at intracavernous carotid or V4 segment of vertebral artery.

**Figure 1:**
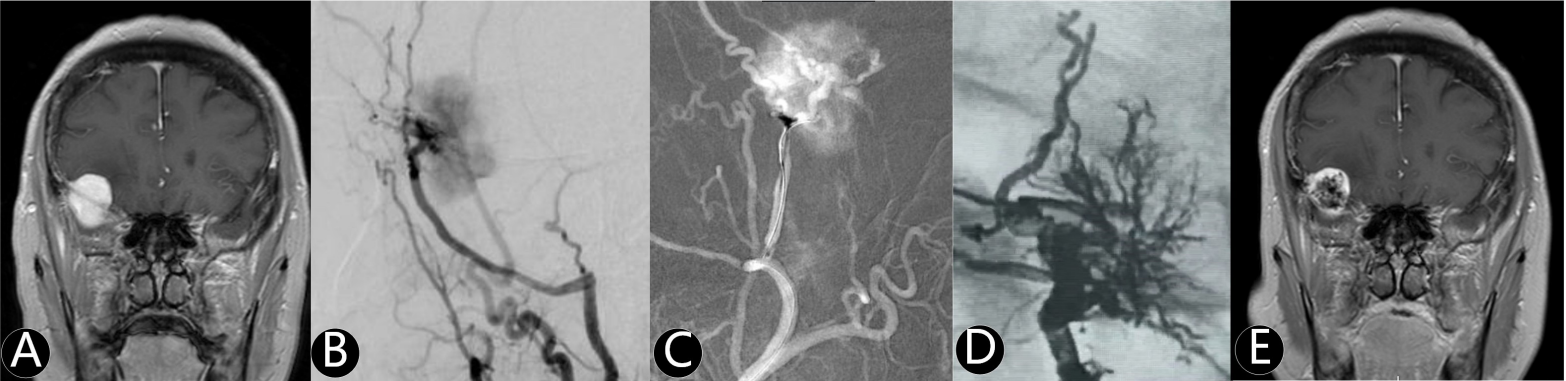
The patient was found right frontotemporal mass by accident. **(A)** Enhanced MR imaging showed right sphenoidal ridge mass with 28.7 cm3. **(B)** The middle meningeal artery was the main supply artery confirmed by cerebral angiography. **(C)** The microcatheter entered the supplying artery and Onyx 18 was injected into the tumor. **(D)** The total tumor (90%) was embolized without craniotomy. **(E)** The volume of tumor reduced at one year after simple embolization.

For the application of Onyx (Medtronic, Minneapolis, MN, USA), the catheter’s dead space was initially filled with dimethyl sulfoxide (DMSO), then Onyx was administered under subtracted road map. Onyx injection was carried out at a very gradual pace, at a rate of 1ml per minute. Techniques to enhance distal penetration while preventing reflux included the “plug and push” method, ensuring sufficient time for forming a proximal plug of Onyx (**Figure 1**). Sometimes, it was useful to create a plug by embolism at end of microcatheter, facilitating anterograde injection-”pressure cooker technology”. APOLLO tip can be left in the tumor bearing vessel.

Glubran 2 (GEM Srl, Viareggio, Italy) was chosen for certain cases. Post-deionization with 5% glucose sugar water after washing gloves, the catheter’s dead space was filled with glucose sugar water. Glubran 2 was then diluted with lipiodol to a concentration of 20%~40% for slow injection into the target arteries (8).

In instances where dangerous anastomoses and lateral branches were challenging to distinguish, a minimal quantity of gelatin sponge was administered slowly through the microcatheter to lessen the risk of irreversible post-embolization complications. Embolization was continued until a desired degree of tumor penetrance or reflux along the microcatheter was reached. A final post-embolization angiogram was performed to ensure there was no embolization in normal cerebral anastomosis (9). Based on the result of embolization; 0: no change 1: partial (50%), 2: subtotal (50–90%), 3: total (>90%), as evaluated by two or more neuro-interventionists.

### HS surgery scheme and/or craniotomy

**Figure 2:**
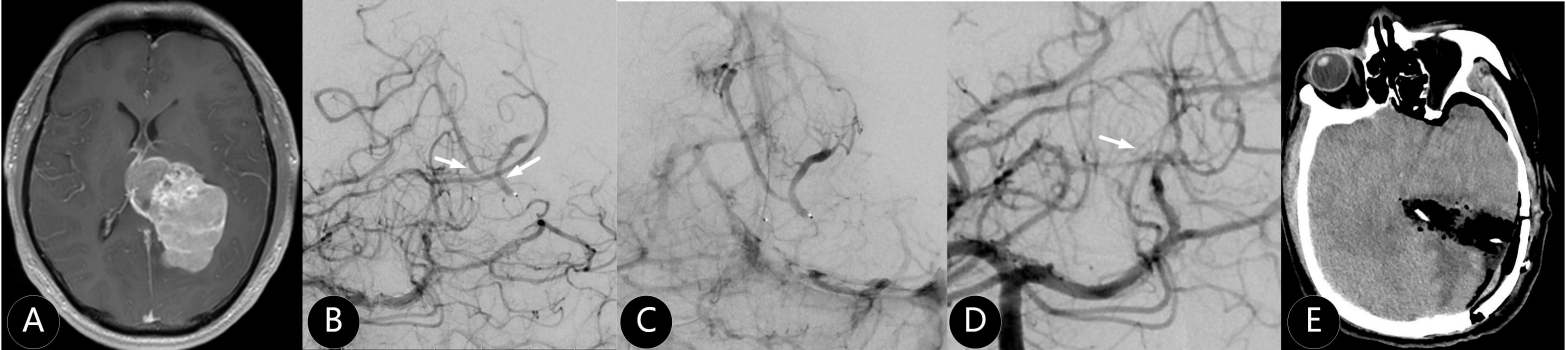
The patient suffered from severe headache for two weeks. **(A)** Enhanced MR imaging showed left paraventricular tumor with 120 cm^3^. **(B, C)** The branches of posterior cerebral artery and superior cerebellar artery were the main supplying arteries. **(D)** One supplying artery was embolized (white arrow) by Onyx 18. **(E)** The meningioma was completely removed and WHO III grade was confirmed by pathology.

The treatment protocol was decided by the neurosurgeons in neurosurgical department. The neurosurgeons who performed the craniotomy and the neurointerventional procedure jointly made the decision. Tumor resection was usually performed within 3 days after embolization. **Figure 2** showed a WHO III grade meningioma was removed totally two days after embolization. The information including tumor size (volume), feeder vessels, the surgery time, hardness of the tumor, blood loss, blood loss per unit volume (ml/ml^3^), the degree of operational incision (Simpson grade), regrowth rate, and prognosis were recorded.

### Postoperative imaging and functional evaluation

CT or MRI scans were conducted at one to five days after embolization and/or surgical procedures to evaluate postoperative changes of tumor and complications such as stroke and edema. The long-term results were evaluated during clinic visits post-surgery, spanning from 9 months to 7 years. Patient status was assessed using the modified Rankin Score (mRS) during follow-up.

### Statistical analysis

All dates were analyzed by SPSS 23.0 software. The normal distribution was calculated by Kolmogorov Smirnov normality test. Those with normal distribution were represented by mean ± standard deviation ( $\bar{\text{x}}$ ± s). were calculated by independent sample T test between two groups. Those with non-normal distribution were represented by median (interquartile distance) [M (Q)], and were calculated by the Mann Whitney U test between groups. Categorical variables were expressed by frequency, and χ2 test was used for comparison between groups. P < 0.05 was considered as the difference.

## Results

### Clinical baseline

A total of 147 patients were enrolled across respective SE group (n=16), HS group (n=67), and control group (n=65). Baseline demographics, including age, sex, and tumor location, were comparable across all groups (**Table 1**; *P*>0.05 for all comparisons).

**Table 1:** Clinical data of Hybrid surgery group, control group and SE group.

	HS group (n= 67)	Control group (n=65)	Test value	P value^*^	SE group (n=16)	Test value	P value^#^
Age	57.5 ± 11.0	60.4 ± 8.0	-1.181	0.237^a^	60.3 ± 10.8	-0.109	0.913 ^a^
Gender	20 (29.9%)	25 (38.5%)	0.732	1.832^b^	5(31.3%)	0.632	1.532^b^
Surgery time	4.9 (2.0)	4.7 (1.8)	-0.845	0.434	–	–	–
Tumor volume (m^3^)	68.8 ± 10.6	35.7 ± 11.3	17.329	<0.001^a^	24.7 ± 5.2	16.126	<0.001^a^
Blood loss (ml/m^3^)	9.8 ± 2.3	19.2 ± 6.5	-11.126	<0.001^a^	–		
Location			7.166	0.127^c^		5.183	0.269^c^
Convexity	47 (70.1%)	30 (46.2%)			6 (37.4%)		
Parasagittal	10 (14.9%)	19 (29.2%)			5 (31.3%)		
Sphenoidal ridge	9 (13.4%)	11 (16.9%)			5 (31.3%)		
Lateral ventricle	1 (1.5%)	1 (1.5%)			0		
Tentorial	7 (10.4%)	5 (7.7%)			0		
Simpson grade			9.385	0.025^c^			
1	47 (70.1%)	30 (46.2%)					
2	10 (14.9%)	19 (29.2%)			–		
3	9 (13.4%)	11 (16.9%)			–		
4	1 (1.5%)	5 (7.7%)			–		
WHO grade			2.667	0.264^c^			
1	50 (74.6%)	49 (75.4%)			–		
2	14 (20.9%)	9 (13.8%)			–		
3	3 (4.5%)	7 (10.8%)			–		
Pe-operational mRS			0.876	0.831^c^		70.642	<0.001^c^
0	4 (6.0%)	3 (4.6%)			14 (87.5%)		
1	49 (73.1%)	52 (80.0%)			2 (12.5%)		
2	11 (16.4%)	8 (12.3)			0		
3	3 (4.5%)	1 (1.5%)			0		
Feeder						2.715	0.257^c^
ICA	8 (11.9%)	–	–	–	0		
ECA	57 (85.1%)	–	–	–	16 (100.0%)		
VA	2 (3.0%)	–	–	–	0		
Material						0.073	0.787^b^
Onyx	48 (71.6%)	–	–	–	12 (75.0%)		
Glubran 2	18 (26.9%)	–	–	–	4 (25.0%)		
Gelatin sponge	1 (1.5%)						
Devascularization rates						9.555	0.008^c^
partial (50%)	13 (19.4%)	–			0		
subtotal (50–90%)	37 (55.2%)	–			7 (43.8%)		
total (>90%)	17 (25.4%)	–			9 (56.3%)		
Immediate Complication^1^	2 (3.0%)				1 (6.3%)	0.327	0.657^b^
Immediate Complicaiton^2^	15 (22.4%)	20 (30.8%)	1.189	0.275^b^	–		
Improved after one year			0.158	0.924^c^		2.157	0.341^c^
No change	53 (79.1%)	51 (78.5%)			15 (93.8%)		
Deteriorate	8 (11.9%)	9 (13.8%)			0		
Improved	6 (9.0%)	5 (7.7%)			1 (6.3%)		
Rumor recurrence	5 (7.5%)	4 (6.2%)	0.1833	0.437^b^	2 (12.5%)	0.424	0.515

^a^T value; ^b^ χ^2^ value; ^c^Z value; HS, hybrid surgery, SE, single embolism; “*”: the data of control group compared with CS group; “#”: the data of SE group compared with HS group. Immediate Complication^1^: the immediate complication after embolism; immediate Complication^2^: the immediate complication after tumor excision.

### HS group and control group

**Figure 3:**
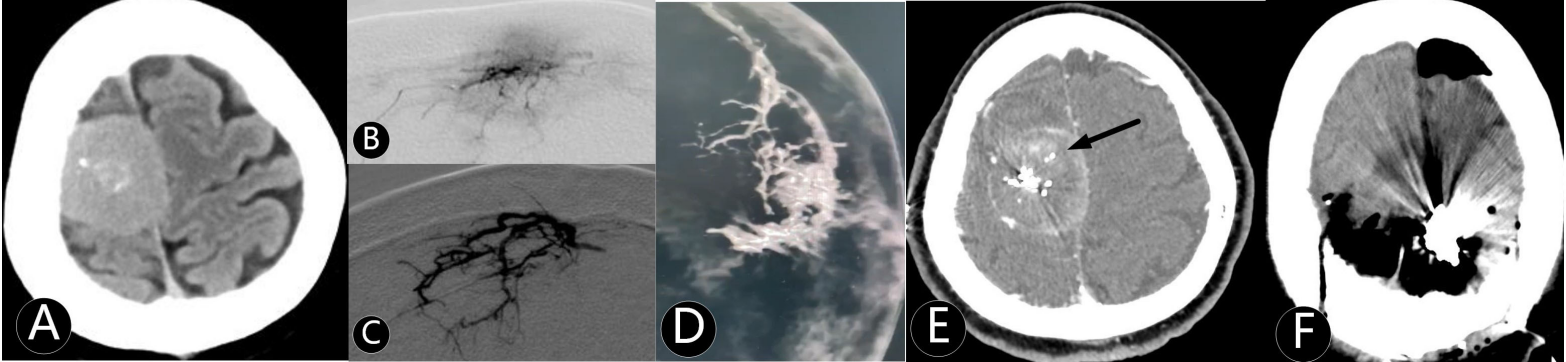
The patient was admitted several times for seizures. **(A)** CT showed right parasagittal mass with 75 cm^3^. **(B)** Microcatheter imaging showed the tumor outline. **(C–E)** The tumor was embolized subtotal by Onyx 18. **(F)** The mass was completely removed at second day after embolization.

The HS group (**Figure 3**) exhibited significantly larger tumor volumes (68.8 ± 10.6 cm³ vs 35.7 ± 11.3 cm³; *P*<0.001) but demonstrated reduced intraoperative blood loss per tumor volume (9.8 ± 2.3 mL/cm³ vs 19.2 ± 6.5 mL/cm³; *P*<0.001) and higher gross total resection rates in Simpson 1 grade (70.1% vs 46.2%; *P* = 0.025), compared with control group. No significant differences were observed in baseline neurological function (mRS), operative time, or 30-day postoperative complication rates (*P*>0.05).

### SE group and HS group

Patients in the SE group had smaller tumors (24.7 ± 5.2 cm³ vs. 68.8 ± 10.6 cm³; *P*<0.001), better baseline neurological function (median mRS: 0 vs. 1; *P*<0.001), and achieved higher total angiographic devascularization rate (56.3% vs. 25.4%; *P* = 0.008). All embolized tumors in the SE group were exclusively accessed through ECA system.

### Mortality and complications

One patient (1.5%) died of pulmonary infection two weeks postoperatively in HS Group. One patient (1.5%) experience severe ischemic stroke and refractory status epilepticus one month after surgery in control group. The cumulative overall mortality rate at 2-year follow-up was 3.08% (2/65 in control group, 1/67 in HS group, 0/15 in SE group), with no additional stroke-related deaths observed during follow-up.

At final follow-up (median: 32 months), recurrence rates were 7.5% (5/67) in HS group, 6.2% (4/65) in control group and 12.5% (2/15) in SE Group, respectively, and statistically significant differences were observed in recurrence rates across groups. Similarly, changes in neurological function (mRS from baseline to follow-up) did not differ significantly between groups.

## Discussion

We demonstrate that the hybrid surgical strategy (preoperative embolization followed by craniotomy) significantly reduced intraoperative blood loss and improved complete resection rate particularly for large tumors. Simultaneously, standalone embolization emerged as a feasible option for small, ECA-supplied meningiomas, achieving satisfying devascularization with a lower complication rate.

The risk of meningioma surgery remains significant and cannot be overlooked. Age, comorbidities, intraoperative operations and decisions and postoperative complications all can significantly influence outcomes (10, 11). Preoperative embolization has been demonstrated to be a reasonable adjunct to resection for appropriately selected intracranial meningiomas and several previous studies supported preoperative embolization in reducing transfusion requirements (12, 13). Because of subjectivity in treatment, a meta-analysis of 1,782 patients found no significant differences in blood loss, operative time, or complications between embolized and non-embolized cohorts (14). These discrepancies likely derived from variability in feeding arteries, embolic endpoints, tumor size and location. Critics further highlight risks of embolization for meningiomas such as radiation exposure, contrast nephropathy, and inadvertent occlusion of critical anastomoses (15, 16). In this study, meningiomas in HS group were predominantly located in the convexity and parasagittal regions, accounting for 70.1% and 14.9%, respectively, which were mainly supplied by the ECA system. Embolization was considered comparatively safer in these two regions due to the lower prevalence of dangerous anastomoses. We found that compared to control group, patients in HS group had larger meningioma volumes but exhibited less intraoperative bleeding per unit volume and achieved a higher extent of resection, indicating that preoperative embolization is effective and beneficial for tumor resection. Future prospective and controlled studies are necessary to ascertain the influences of preoperative embolization of intracranial meningiomas with respect to extent of resection, operative duration, operative blood loss, and surgical complicatons (17).

Embolization of ICA or VA-supplied meningiomas remains challenging due to vessel tortuosity and proximity to core functionality (18). Embolization via the ICA and VA routes was undertaken with particular caution, accounting only for 12.0% (10/83), including six through ophthalmic artery (OphA) for anterior cranial base meningiomas, two through anterior cerebral artery for convex meningioma, and two through the posterior cerebral artery for intraventricular meningioma. Although technically viable, embolization performed via OphA carries a significant risk of visual complications, precluding its routine recommendation. The current evidence base is insufficient to support widespread adoption of this technique (19). At our institution, embolization via OphA accounted only for 6.0% (4/67) of cases, which was not our usual options, because of high risks of its origin of the central retinal artery. Procedural hazards like arterial dissection or inadvertent reflux of embolic material into the central retinal artery during super-selective microcatheterization and embolization significantly elevate the potential for permanent vision loss (20). Super-selective provocative testing to assess neurological risk could be a solution (21, 22), however, all of our patients are under general anesthesia, making it difficult to carry out. 3D rotational DSA-guided pressure cooker technique can be an effective and safe way for precise tumor-normal vessel demarcation (23, 24). In our two cases through OphA embolization, Apollo microcatheters were super-selectively advanced to distal branches and proximal plugs were established using coils combined with Onyx-18, followed by predominant tumor bed devascularization achieved with additional Onyx injection. For our one case of posterior cerebral artery embolization in HS group, where ventricular meningioma supply could not be clearly distinguished from normal perforators, temporary flow reduction was achieved using gelatin sponge particles, as Bendszus’ technique shown (25).

Approximately 20–25% of patients with small-volume meningiomas experienced tumor growth and developed related symptoms during follow-up (26, 27). A considerable number of these patients also opted for observational follow-up at our two neurosurgical centers. For those who required intervention, we provided treatment options including craniotomy and interventional therapy, allowing patients and their families to make an informed decision after thorough discussion. In cases where angiography identified arterial supply to the tumor, we recommended embolization of the tumor-feeding arteries. All meningioma patients undergoing SE presented without mass effect and associated clinical symptoms. Among these, 9 cases achieved anatomical total devascularization characterized by the arborized intratumoral penetration sign of embolic agents and were advised surveillance follow-up. The remaining 7 subtotal cases (embolization extent 60-90%) opted for surveillance after multidisciplinary consultation, where patients or families made informed decisions following comprehensive disclosure of therapeutic alternatives. Aihara et al. also pointed out that small size, superficial location, and absence of pial supply were independent factors for total devascularization (28). In our cohort, compared to the HS group, SE patients had smaller tumor volumes with minimal neurological deficits. 87.5% (14/16) achieved mRS 0 with no deficit, while the remaining two had mRS 1, including a 67-year-old with prior stroke history and another diagnosed post-traumatic brain injury, neither presenting symptoms attributable to their meningiomas. Notably, 93.8% (15/16) of small ECA-supplied tumors showed radiographic stabilization or regression over 24 months, suggesting blood flow deprivation may induce tumor, being similar with Sluzewski’s six patients by sole embolization therapy (22). Even so, extended surveillance is critical to detect delayed recurrence (29). Even if renewed growth occurs, these tumors can still be treated via craniotomy resection.

Embolizing branches of the ECA generally poses fewer technical challenges and lower risks compared to targets via the ICA. However, it still carries potential neurological risks, especially in small tumors with minimal blood supply. We believe greater caution should be exercised when embolizing asymptomatic small skull base meningiomas. Performing super-selective catheterization is necessary to target feeding vessels while preserving normal functional vascular anastomoses. The vascular supply to cranial nerves VII and IX-XII partially derives from ECA tributaries, including the petrosal branch of the middle meningeal artery (MMA), stylomastoid branch of the posterior auricular artery, and the branches of the ascending pharyngeal artery. Embolization through these arteries or their branches risks injury to these cranial nerves (30). Furthermore, pre-existing ECA-ICA anastomoses create pathways for inadvertent intracranial embolization and stroke (31–33). Notably, OphA may alternatively arise from the MMA, and failure to identify this variant during MMA embolization can also lead to inadvertent central retinal artery occlusion with subsequent blindness (34, 35).

Among all embolization cases, Onyx was utilized in 72.3% patients (60/83) primarily due to its superior deep tumoral penetration capability that enabled extensive vascular network filling while controlling reflux (36). Conversely, Glubran 2 was employed in 22 cases where rapid occlusion of dominant feeding pedicles was prioritized, though this required precise injection to prevent non-target embolization (37). The selection between these two embolic agents can be tailored based on specific tumor characteristics and the operator’s comprehensive clinical judgment.

## Limitation

This study is inherently limited by its retrospective design, small cohort, and short-term follow-up across two neurosurgical centers, which may underpower statistical analyses and preclude definitive conclusions about complication or risks long-term recurrence. Future prospective multicenter trials, incorporating standardized embolization grading systems and stratified randomization by tumor location and/or feeder type, are warranted to validate the efficacy of preoperative or standalone embolization, particularly for rare subtypes, such as petroclival, cavernous sinus.

## Conclusion

This multicenter study validates the role of preoperative embolization in optimizing large meningioma resection and identifies standalone embolization as a potential curative option for small ECA-supplied tumors. Our angiographic grading system and anatomy-driven protocol provide tools for personalized care. Future prospective trials should integrate novel embolic agents and advanced imaging to refine subtype-specific strategies and validate long-term outcomes.

## Data Availability

The raw data supporting the conclusions of this article will be made available by the authors, without undue reservation.
